# Widespread loss of the silencing epigenetic mark H3K9me3 in astrocytes and neurons along with hippocampal-dependent cognitive impairment in C9orf72 BAC transgenic mice

**DOI:** 10.1186/s13148-020-0816-9

**Published:** 2020-02-18

**Authors:** Nur Jury, Sebastian Abarzua, Ivan Diaz, Miguel V. Guerra, Estibaliz Ampuero, Paula Cubillos, Pablo Martinez, Andrea Herrera-Soto, Cristian Arredondo, Fabiola Rojas, Marcia Manterola, Adriana Rojas, Martín Montecino, Lorena Varela-Nallar, Brigitte van Zundert

**Affiliations:** 1 Institute of Biomedical Sciences (ICB), Faculty of Medicine & Faculty of Life Sciences, Universidad Andres Bello, Santiago, Chile; 2 CARE Biomedical Research Center, Faculty of Biological Sciences, Pontificia Universidad Católica de Chile, Santiago, Chile; 3 FONDAP Center for Genome Regulation, Santiago, Chile; 4 Current address: Faculty of Health Sciences, Universidad Autónoma de Chile, Santiago, Chile; 5 Program of Human Genetics, ICBM, Faculty of Medicine, Universidad de Chile, Santiago, Chile; 6 Instituto de Genética Humana, Pontificia Universidad Javeriana, Bogotá, Colombia

**Keywords:** H3K9me3, ALS, FTD, Brain, Astrocyte, Neuron, Memory

## Abstract

**Background:**

Hexanucleotide repeat expansions of the G_4_C_2_ motif in a non-coding region of the *C9ORF72* gene are the most common genetic cause of amyotrophic lateral sclerosis (ALS) and frontotemporal dementia (FTD). Tissues from C9ALS/FTD patients and from mouse models of ALS show RNA foci, dipeptide-repeat proteins, and notably, widespread alterations in the transcriptome. Epigenetic processes regulate gene expression without changing DNA sequences and therefore could account for the altered transcriptome profiles in C9ALS/FTD; here, we explore whether the critical repressive marks H3K9me2 and H3K9me3 are altered in a recently developed C9ALS/FTD BAC mouse model (C9BAC).

**Results:**

Chromocenters that constitute pericentric constitutive heterochromatin were visualized as DAPI- or Nucblue-dense foci in nuclei. Cultured C9BAC astrocytes exhibited a reduced staining signal for H3K9me3 (but not for H3K9me2) at chromocenters that was accompanied by a marked decline in the global nuclear level of this mark. Similar depletion of H3K9me3 at chromocenters was detected in astrocytes and neurons of the spinal cord, motor cortex, and hippocampus of C9BAC mice. The alterations of H3K9me3 in the hippocampus of C9BAC mice led us to identify previously undetected neuronal loss in CA1, CA3, and dentate gyrus, as well as hippocampal-dependent cognitive deficits.

**Conclusions:**

Our data indicate that a loss of the repressive mark H3K9me3 in astrocytes and neurons in the central nervous system of C9BAC mice represents a signature during neurodegeneration and memory deficit of C9ALS/FTD.

## Background

Amyotrophic lateral sclerosis (ALS) is a lethal disease characterized by extensive degeneration of cranial, brainstem, and spinal motoneurons in adulthood. Frontotemporal dementia (FTD) is a progressive neuronal atrophy in the frontal and temporal cortices and is characterized by progressive deficits in behavior, language, and executive function [1, 2]. FTD is the second most common cause of early dementia (< 65 years), after Alzheimer’s disease [3]. ALS and FTD share similar genetic signatures, including mutations in *TDP-43* and *C9ORF72* [3–5]. Moreover, patients harboring mutations in *C9ORF72* may suffer from ALS, FTD, or a combination of the two, which explains the wide clinical diversity of the two diseases [6].

Hundreds to thousands of hexanucleotide repeat expansions of the G_4_C_2_ motif in a non-coding region of the *C9ORF72* gene (intron 1) are now regarded as the most common genetic cause of ALS and FTD, referred to as C9ALS/FTD [7, 8]. Analyses of postmortem brain tissues of C9ALS/FTD patients, as well as of patient-derived cultured cells, have led to proposed mechanisms whereby *C9ORF72* repeat expansions cause the diseases; these include loss of C9ORF72 function (i.e., haploinsufficiency) and gain-of-toxicity from repeat-containing RNAs and aberrant dipeptide-repeat (DPR) proteins, through repeat-associated non-AUG-dependent (RAN) translation [4, 5, 9, 10]. To elucidate the disease mechanism(s) associated with C9ALS/FTD, transgenic mice have been generated in which one or both *C9orf72* alleles were inactivated [11], or in which hundreds (≥ 450) of patient-derived G_4_C_2_ hexanucleotide repeat expansions were expressed through bacterial artificial chromosomes (BACs) [11–14]. Unlike the *C9orf72* null mice, all C9BAC mice display the molecular abnormalities that are characteristic of C9ALS/FTD patients, namely, RNA foci and DPRs, which strongly suggest that gain-of-toxicity, and not loss-of-function, is critical for C9ALS/FTD. In addition, transcriptome analyses reveal a large number of aberrantly expressed genes (up- and downregulated) in the cortex of C9BAC mice [12] and in the cortex and hippocampus of a recent engineered mouse model expressing only proline-arginine (PR) DPRs (poly-PR mice) synthesized from expanded G_4_C_2_ repeats [15]. Widespread transcriptome alterations have also been found in diverse brain areas (i.e., frontal cortex, motor cortex, and cerebellum) of postmortem C9ALS/FTD patients, in induced pluripotent stem cell (iPSC)-derived neurons, and in fibroblasts derived from these patients [16–19]. Nevertheless, the mechanistic basis for these alterations has not been established. Here, we investigated whether epigenetic processes are aberrant in C9BAC mice that can account for changes in the expression profile reported in C9ALS/FTD.

Of the two major types of chromatin, euchromatin corresponds to a relaxed and transcriptionally active chromatin conformation, while heterochromatin is characterized by a condensed and transcriptionally silent organization [20, 21]. Heterochromatin is further classified into facultative and constitutive forms. Facultative heterochromatin (fHC) comprises regions containing genes that are differentially expressed throughout development and/or differentiation and which then become silenced. Conversely, constitutive heterochromatin (cHC) is largely formed at pericentromeres and telomeres that are gene-poor regions that mainly contain repetitive sequences, including transposable elements as well as tandemly arranged simple or satellite repeats [20, 22]. To regulate the compaction of HC, the nucleosomal histones in the HC regions are enriched by specific epigenetic marks. In particular, cHC is characterized by relatively high levels of the trimethylated form of lysine 9 of histone H3 (H3K9me3), while the fHC is enriched for H3K9me2; these H3K9me2/me3 marks repress gene transcription, maintain genome stability (by silencing repetitive DNA elements and transposons), and protect DNA from damage [20, 21, 23–27]. Recent studies document that the distributions and/or expression levels of H3K9me2/me3 are altered in the brains of patients and also in models of Alzheimer’s disease [26, 28–30], Huntington’s disease [31], and Rett syndrome [32].

Here, we explore the nuclear distribution and expression levels of the repressive H3K9me2/me3 and neutral H3K9me1 marks in tissue and cultures from the recently generated mouse model C9BAC [13]. We report that the staining intensity of H3K9me3 (but not of H3K9me2 or H3K9me1) at chromocenters is markedly reduced in primary astrocytes from these mice. A similar reduction in H3K9me3 signal at chromocenters is observed in astrocytes and neurons in the spinal cord, motor cortex, and hippocampus of C9BAC mice. The alteration in epigenetic histone marks at C9BAC hippocampi parallels a previously undetected loss of neurons in CA1, CA3, and dentate gyrus, and a hippocampal-dependent cognitive deficit in the C9ALS/FTD mouse model. Our results suggest that the loss of H3K9me3 plays a relevant role in the mechanistic underpinnings of neurodegeneration and cognitive deficits in C9BAC mice.

## Results

### Reduced levels of nuclear H3K9me3 in primary spinal cord astrocytes from C9BAC mice

To explore the role of mono-, di-, and tri-methylation marks of histone H3 (H3K9me1/me2/me3) and histone modifications in the C9orf72 pathology, we first assessed the subnuclear distribution of these marks in primary cultures of spinal cord astrocytes derived from the recently engineered C9BAC mice [13]. We particularly focused on astrocytes as multiple studies have shown that these glial cells and astrocyte-derived conditioned media (ACM) from cultured mouse and human fALS (mutSOD1, mutTDP43, and mutC9orf72) and sALS astrocytes induce non-cell autonomous toxicity towards motoneurons by releasing soluble neurotoxic factor(s) [33–37]. In line with these studies, we found that ACM from C9BAC astrocytes also significantly reduced motoneuron survival, by ~ 30% relative to ACM from control mice (wild-type non-transgenic littermates) (Additional file 1: Figure S1).

To evaluate the pattern of nuclear distribution and intensity of H3K9me3 mark in C9BAC and control astrocytes, we used immunostaining with a widely used commercial antibody against this modification [38–40]. Additionally, we tested the specificity of the H3K9me3 staining in N2A cells treated with chaetocin, a pharmacological inhibitor of H3K9 methyltransferase SUV39H1/2. As expected [41], a complete loss of the nuclear H3K9me3 signal was observed in cells treated with chaetocin (Additional file 1: Figure S2).

The nuclear stain DAPI was used to evaluate the global chromatin organization, particularly of the cHC that is located pericentrically. This region consists of very large arrays of tandemly repeating, non-coding DNA sequences termed major satellite DNA repeats. The AT-rich 234-bp long major satellite repeats may extend to more than 2 Mb per chromosome and are preferentially revealed with fluorescent DNA stains such as DAPI, TO-PRO-3, NucBlue, and Hoechst. In interphase and differentiated mouse nuclei, pericentric cHC clusters into large structures known as chromocenters, which are specifically marked by H3K9me3, giving rise to H3K9me3-positive foci [20, 26, 42–44].

**Fig. 1:**
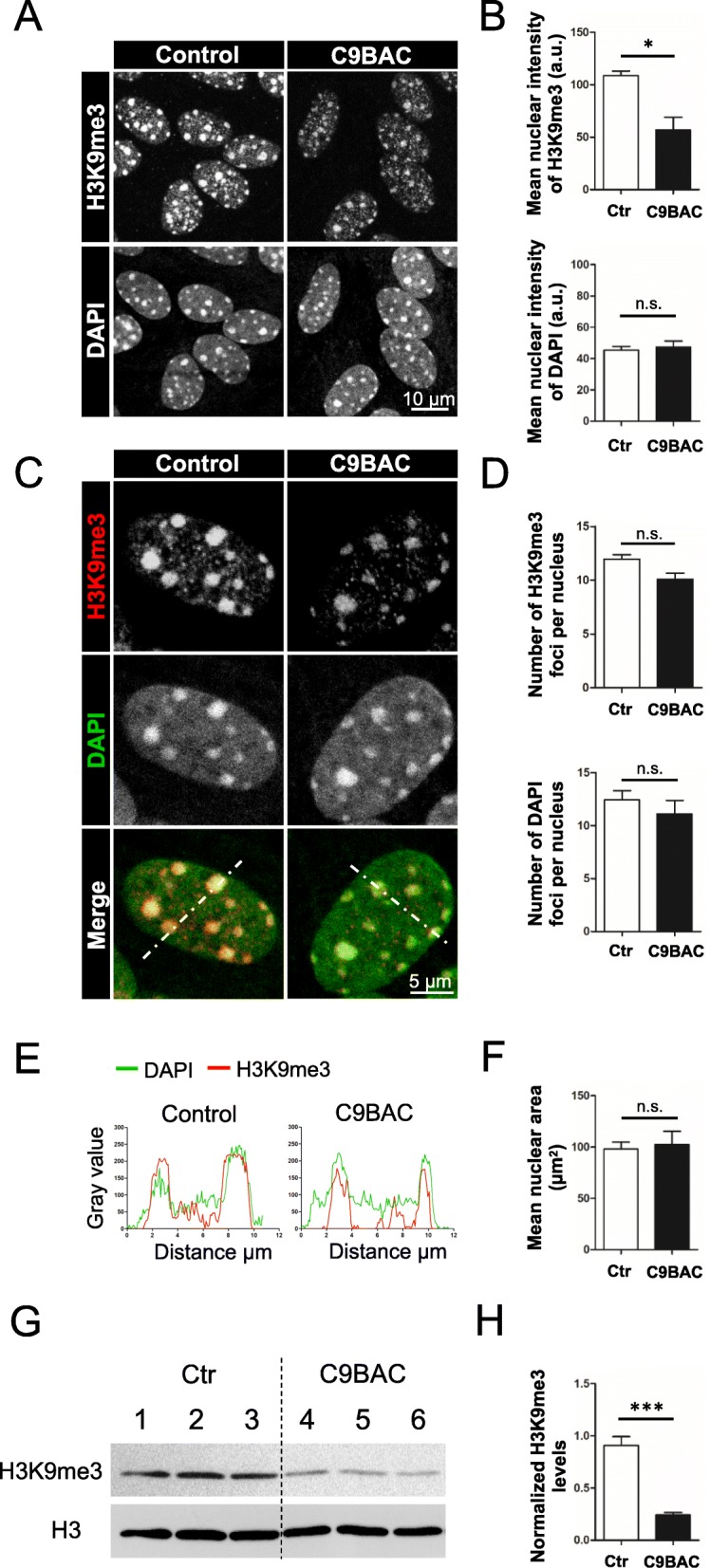
Reduced H3K9me3 staining at chromocenters in primary cultured astrocytes from C9BAC mice is accompanied by global loss of the H3K9me3 mark. **a** Representative confocal images of the immunofluorescence staining of H3K9me3 in primary cultures of control and C9BAC astrocytes. Nuclei are stained with DAPI. The images represent a maximum projection for the total nuclear volume. **b** Quantification of the mean nuclear signal staining intensity of H3K9me3 (upper graph) and DAPI (lower graph) shown as arbitrary units (a.u.). **c** Higher magnification of nuclei from control and C9BAC astrocytes immunostained for H3K9me3 (white and red) and stained for DAPI (white and green) are shown individually and merged. Images are single confocal sections. **d** Quantification of the number of H3K9me3-positive (upper graphs) and DAPI-positive foci (lower graphs) per nucleus. **e** Quantification of the fluorescence intensity of H3K9me3 (red) and DAPI (green) in a line scan drawn across chromocenters in nuclei from control and C9BAC astrocytes using a single confocal section. **f** Quantification of the nuclear area (μm^2^). In all graphs, bars represent mean ± SEM. *P< 0.05; non-statistical differences (ns), Student’s *t* test (*n* = 3 independent experiments, at least 30 cells were analyzed per condition in each experiment). **g** Western blot analysis of H3K9me3 from total nuclear lysates of control (lanes 1–3) and C9BAC (lanes 4–6) astrocytes from three independent experiments are shown. H3 was used as loading control. **h** Densitometric analysis of the western blot in **g** with H3K9me3 normalized to total H3 levels. Bars represent mean ± SEM. ***P< 0.001, Student’s *t* test

Confocal images showed that primary astrocytes from control mice exhibit abundant bright large (≥ 0.5 μm^2^) H3K9me3 foci (Fig. 1a; Additional file 1: Figure S3). Astrocytes derived from C9BAC mice also displayed H3K9me3 foci; however, image analysis and quantification revealed that the mean H3K9me3 signal intensity per nucleus was significantly reduced in C9BAC astrocytes compared with control astrocytes (Fig. 1a, b; Additional file 1: Figure S3). The reduction of the mean nuclear intensity of H3K9me3 staining in C9BAC astrocytes was due to the reduced intensity of both the foci and diffuse H3K9me3 staining (Fig. 1c, e); however, the number of H3K9me3 foci per nucleus was found unchanged (Fig. 1d), and these foci still co-localized with DAPI-chromocenters (Fig. 1e). In C9BAC astrocytes, the total number of DAPI-chromocenters (Fig. 1d) and their mean nuclear area (Fig. 1f) were similar to those found in control astrocytes; additionally, no signs of nuclear blebs were observed (Fig. 1a, c). These results indicate that the loss of H3K9me3 foci in C9BAC primary astrocytes is not accompanied by a robust loss of cHC or of nuclear integrity.

To determine whether the reduction in the nuclear H3K9me3 staining signal was the consequence of a redistribution or a decrease in the total amount of this repressive mark, we carried out immunoblot analysis of H3K9me3 and H3 in nuclear lysates from control and C9BAC astrocytes (Fig. 1g): we found a significant decrease in total levels of H3K9me3 protein relative to control cells (Fig. 1h). These results indicate that the reduced H3K9me3 signal in C9BAC astrocytes involves a global loss of the repressive H3K9me3 modification.

**Fig. 2:**
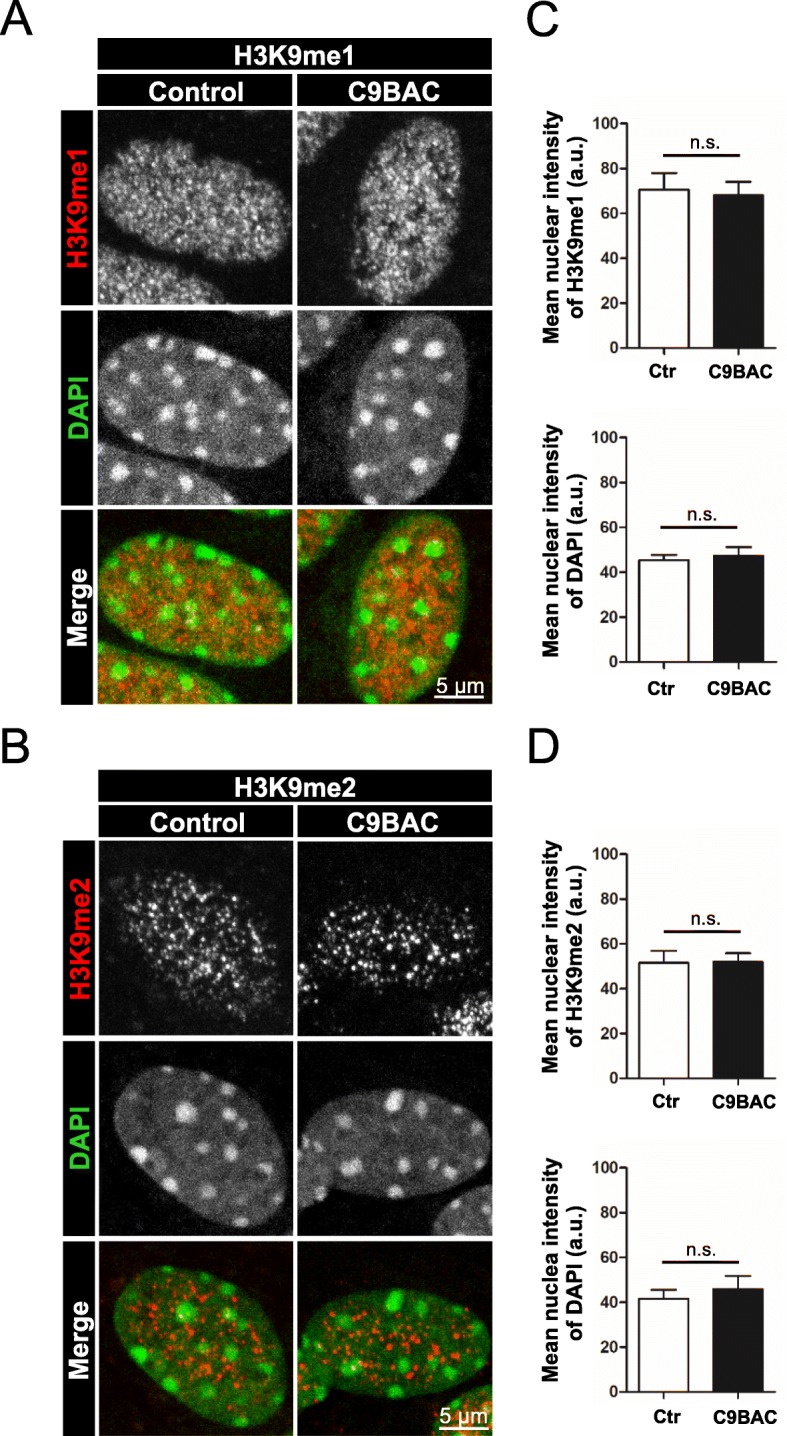
The clustered distribution of H3K9me1/me2 marks is unaltered in cultured astrocytes from C9BAC mice. **a**, **b** Representative confocal images of the immunofluorescence staining of H3K9me1 (**a**, white and red) and H3K9me2 (**b**, white and red) in primary cultures of control and C9BAC astrocytes. Nuclei are stained with DAPI (white and green). Single confocal sections are shown. **c**, **d** Quantification of the mean nuclear intensity (a.u.) of H3K9me1 (**c**, upper graph) and H3K9me2 (**d**, upper graph) staining. Quantification of the mean nuclear intensity of DAPI (a.u.) is also shown (lower graphs). In all graphs, bars represent mean ± SEM. Non-statistical differences (ns), Student’s *t* test (*n* = 3 independent experiments, at least 30 cells were analyzed per condition in each experiment)

We also analyzed H3K9me1 and H3K9me2. In contrast to the H3K9me3 pattern, the immunofluorescence signal for both marks was detected in smaller and blurrier clusters, referred to as puncta. In agreement with previous studies using the same commercial H3K9me2 antibody [45, 46], we found that these puncta did not co-localize with chromocenters in control or C9BAC astrocytes (Fig. 2a, b). No changes in the nuclear distribution or intensity of H3K9me1 (Fig. 2c) and H3K9me2 (Fig. 2d) were observed in C9BAC versus control astrocytes, indicating that these epigenetic marks were not affected.

### Nuclear H3K9me3 staining is reduced in astrocytes and neurons of the spinal cord and motor cortex from C9BAC mice

**Fig. 3:**
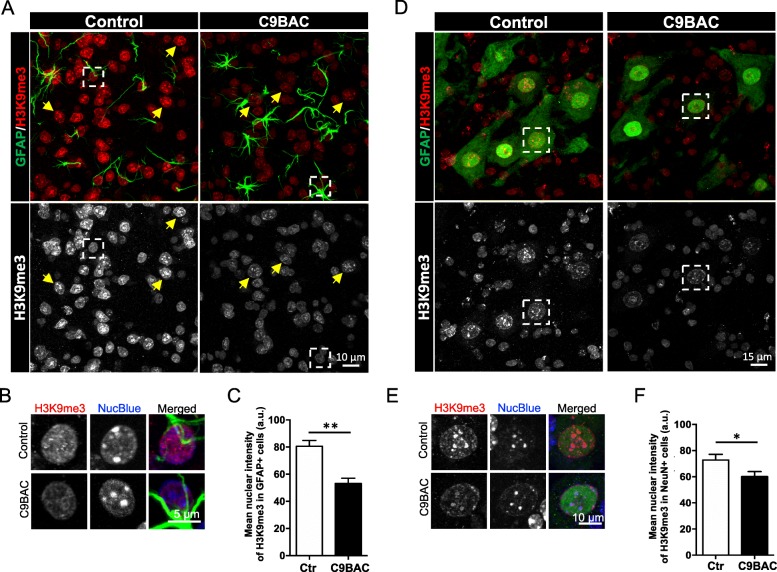
Reduced H3K9me3 staining at chromocenters in spinal cord glial cells and motoneurons from C9BAC mice. **a** Representative confocal images of immunofluorescence staining for H3K9me3 (red and white) and for the activated astrocyte marker GFAP (green) in lumbar spinal cord slices (40 μm) of control and C9BAC mice. The images represent a maximum projection for the total nuclear volume. Arrows mark GFAP-negative cells. Boxes mark selected GFAP-positive cells. **b** GFAP-positive cells selected from the boxed areas in **a** depicted at higher magnifications, showing individual and merged images of H3K9me3 (white and red), NucBlue (white and blue), and GFAP (green). **c** Quantification of the mean nuclear intensity of H3K9me3 staining (a.u.) in GFAP-positive cells. **d** Representative confocal images of immunofluorescence staining for H3K9me3 (red and white) and for the neuronal nuclei marker NeuN (green) in lumbar spinal cord slices (40 μm) of control and C9BAC mice. The images represent a maximum projection for the total nuclear volume. Boxes mark selected NeuN-positive cells. **e** NeuN-positive cells selected from the boxed areas in **d** depicted at higher magnifications, showing individual and merged images of H3K9me3 (white and red), NucBlue (white and blue), and NeuN (green). **f** Quantification of the mean nuclear intensity of H3K9me3 staining (a.u.) in NeuN-positive cells. In all graphs, bars represent mean ± SEM. *P< 0.05, **P< 0.01, unpaired Student’s *t* test (*n* = 3 mice, at least 20 NeuN-positive and 20 NeuN-negative cells were analyzed per animal)

Given the reduced levels of H3K9me3 observed in primary astrocytes, we next determined if similar epigenetic changes occur in the astrocytes and neurons of C9BAC mice. To determine the subnuclear localization of H3K9me3 in astrocytes and neurons of the spinal cord of 9-month-old C9BAC and control (non-transgenic littermates) mice, lumbar spinal cord tissue sections (L4-6) were immunostained for glial fibrillary acidic protein (GFAP) and neuronal nuclei (NeuN) to identify astrocytes and neurons, respectively, in the dorsal and ventral spinal cord. NucBlue was used to stain DNA and to visualize chromocenters. We found that the intensity of GFAP staining signal was found increased in C9BAC spinal cord samples relative to control tissue (Fig. 3a). Confocal images (Fig. 3a, b) and quantification (Fig. 3c) revealed a significant reduction in the intensity of H3K9me3 nuclear staining in C9BAC astrocytes in L4-6 ventral spinal cord, compared with control astrocytes. The H3K9me3 signal in C9BAC mice was strictly nuclear (Fig. 3a, b), and alterations were detected neither in the NucBlue staining pattern nor in the nuclear area; mean NucBlue intensity 98 ± 3 and 102 ± 4 a.u. (*P* value = 0.16) and mean area 49 ± 1 and 52 ± 2 μm^2^ (*P* value = 0.25) for control and C9BAC, respectively. These results show that, as observed for cultured C9BAC astrocytes, H3K9me3 is reduced in astrocytes from L4-6 spinal cord in C9BAC mice, without significant loss of cHC or of nuclear integrity.

Our confocal imaging analysis also revealed prominent H3K9me3 staining in GFAP-negative cells in control mice, and the intensity of this signal was strongly reduced in tissue sections from C9BAC mice (Fig. 3a). To directly determine whether the clustered distribution of H3K9me3 was altered in spinal cord motoneurons from C9BAC mice, we used triple fluorescence staining for H3K9me3, NucBlue, and the neuronal marker NeuN (Fig. 3d). Ventral spinal cord sections (L4-6) from control mice showed numerous NeuN-positive motoneurons that could be readily identified due to their larger soma size (≥ 30 μm) and their specific location within the spinal cord (Fig. 3d); C9BAC samples did not display evident alterations in the number or size of NeuN-positive motoneurons (Fig. 3d). These results are in agreement with the initial characterization of the C9BAC mouse model that reported the absence of behavioral changes or histological abnormalities in the motor system [13]. Similar to glial cells, the mean staining intensity of H3K9me3 was significantly reduced in the nuclei of NeuN-positive motoneurons of the C9BAC mice (Fig. 3e, f).

The staining pattern of H3K9me3 and NucBlue was markedly different in the nuclei of glial cells (Fig. 3b) versus motoneurons (Fig. 3e) in the spinal cord: specifically, whereas H3K9me3 foci and chromocenters were distributed as prominent round structures throughout the nuclei of astrocytes, H3K9me3 foci and chromocenters were concentrated at the nuclear and nucleolar periphery in control motoneurons. These H3K9me3 foci within perinuclear and/or perinucleolar chromocenters have been especially visible in highly differentiated cells, including mature neurons (i.e., [26]), mature hematopoietic cells [47], and muscle cells [48]. Despite the overall loss of H3K9me3, the staining intensity of NucBlue (56 ± 5 and 61 ± 2 a.u. for control and C9BAC, respectively with a *P* value = 0.77) and the nuclear areas (99 ± 5 and 93 ± 4 μm^2^ for control and C9BAC, respectively with a *P* value = 0.32) were similar for C9BAC and control motoneurons.

**Fig. 4:**
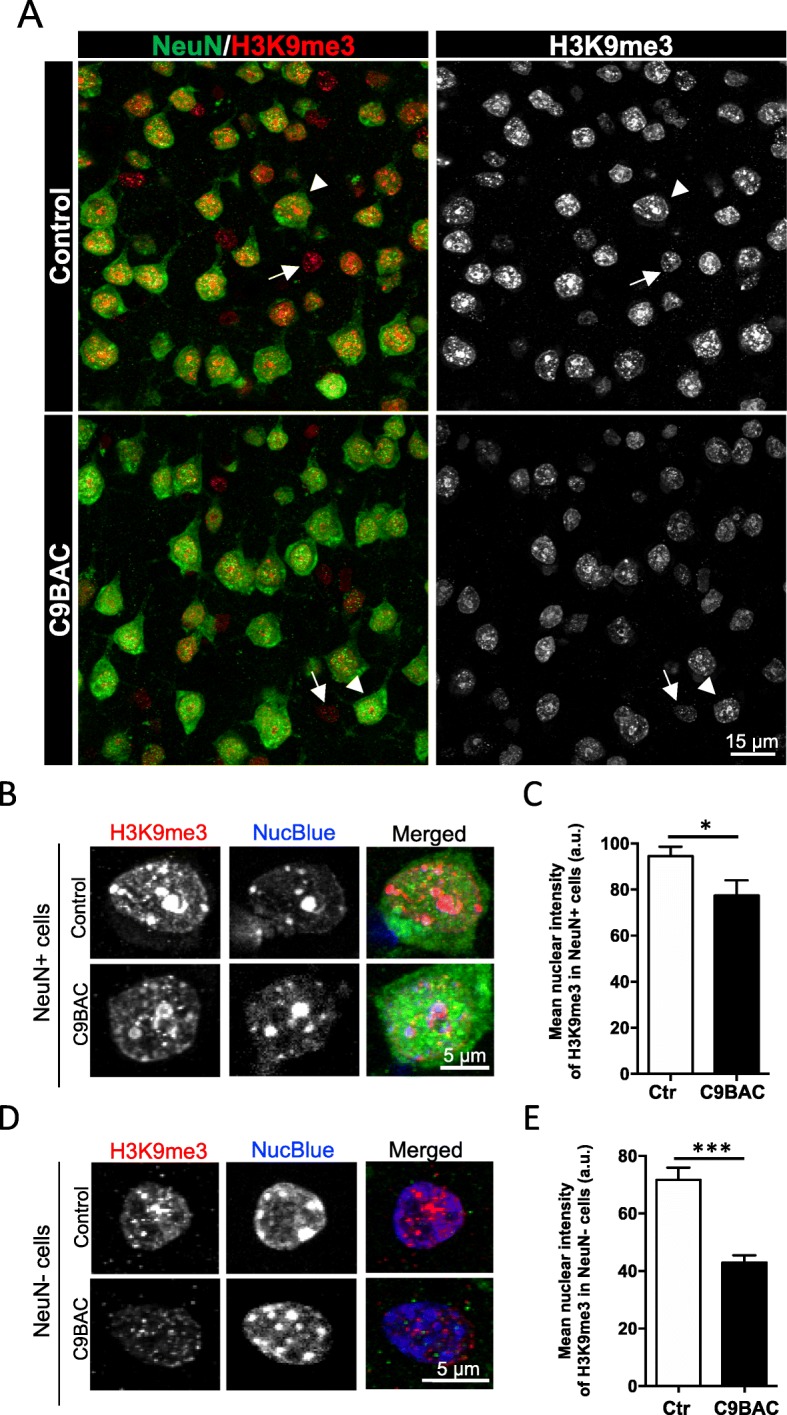
H3K9me3 staining at chromocenters is reduced in neurons of the motor cortex in C9BAC mice. **a** Representative confocal images of immunofluorescence staining for H3K9me3 (red and white) and for NeuN (green) in the motor cortex from control and C9BAC mice (coronal brain section 40 μm). The images represent a maximum projection for the total nuclear volume. Arrowheads indicate selected NeuN-positive cells, and arrows indicate selected NeuN-negative cells. **b**, **d** NeuN-positive (**b**) and NeuN-negative (**d**) cells selected from **a** depicted at higher magnifications, showing individual and merged images of H3K9me3 (white and red), NucBlue (white and blue), and NeuN (green). **c**, **e** Quantification of the mean nuclear intensity of H3K9me3 staining (a.u.) in NeuN-positive (**c**) and NeuN-negative (**e**) cells. In all graphs, bars represent mean ± SEM. *P< 0.05, ***P< 0.001, unpaired Student’s *t* test (*n* = 3 mice, at least 25 NeuN-positive and 15 NeuN-negative cells were analyzed per animal)

The staining pattern and intensity of H3K9me3 were also evaluated in sections through the motor cortex of control and C9BAC mice (Fig. 4a). Images and quantification revealed that the mean staining intensity for H3K9me3 per nucleus was significantly reduced in NeuN-positive (Fig. 4b, c) as well as NeuN-negative (Fig. 4d, e) cells in sections through the motor cortex in C9BAC mice. Loss of H3K9me3 nuclear staining in C9BAC NeuN-positive cells was not accompanied by alterations in NucBlue-chromocenter staining or in the nuclear area (mean NucBlue intensity 87 ± 3 and 93 ± 3 a.u. (*P* value = 0.56) and mean area 116 ± 3 and 124 ± 4 μm^2^ (*P* value = 0.71) for control and C9BAC, respectively) neither in NeuN-negative cells (mean NucBlue intensity 98 ± 4 and 92 ± 3 a.u. (*P* value = 0.68) and mean area 42 ± 2 and 44 ± 3 μm^2^ (*P* value = 0.28) for control and C9BAC, respectively). These data indicate that the reduced nuclear H3K9me3 staining is not accompanied by evidence of impairments in cHC or in nuclear integrity.

### Reduced nuclear staining of H3K9me3 in hippocampal neurons of the C9BAC mouse is accompanied by neuronal loss and OLM deficit

**Fig. 5:**
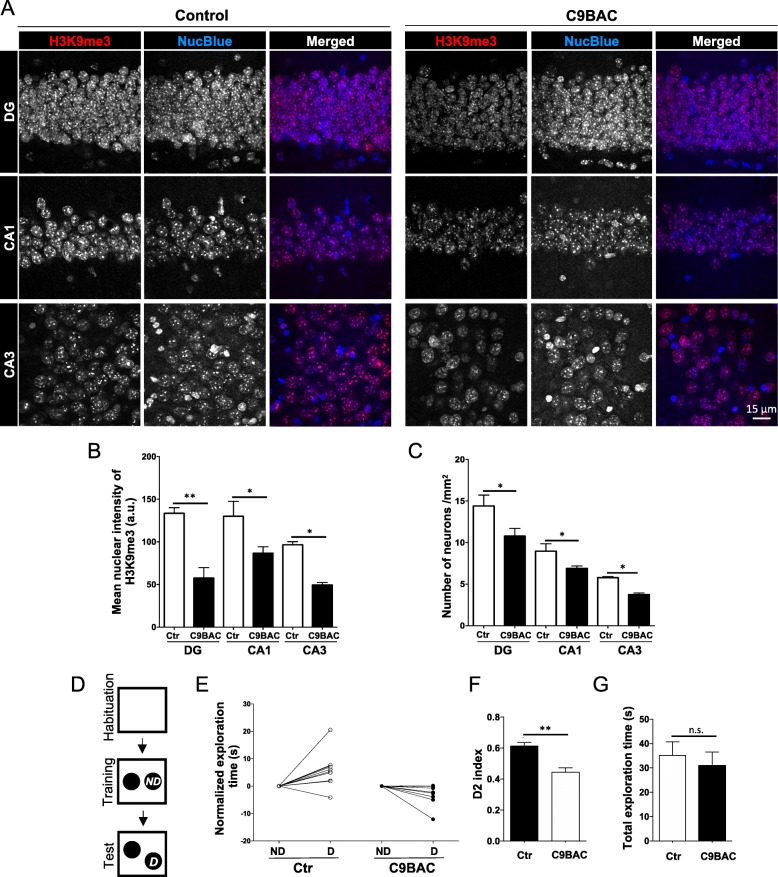
C9BAC mice show reduced intensity of H3K9me3 staining at chromocenters, hippocampal neurons, and deficits in OLM. **a** Representative immunostaining of H3K9me3 in the dentate gyrus (DG), CA1, and CA3 hippocampal regions of control and C9BAC mice (coronal brain section 40 μm). Images show maximum projection. **b** Quantification of mean nuclear intensity of H3K9me3 staining (a.u.) per nucleus (*n* = 3). **c** The density of neuronal nuclei in every region of the hippocampus was quantified (*n* = 4). In the graphs, bars represent mean ± SEM. *P< 0.05, **P< 0.01, one-way ANOVA (at least 30 nuclei were analyzed per animal). **d** Overview of the OLM task, in which mice are subjected to habituation, followed by exposure to two similar objects (“Training”), then tested 24 h later (“Test”) by re-exposure for 10 min to the same testing area with one non-displaced object (ND) and one displaced object (D). **e** Graph shows normalized exploration time (in seconds) spent by individual control (Ctr) and C9BAC mice on non-displaced (ND) versus displaced (D) object. **f** Discrimination index (D2) corresponding to the time spent in exploring the displaced (D) object over the total time exploring both objects: an index of 0.5 indicates that mice did not discriminate between the D and ND object. **g** Total exploration time. Bars represent means ± SEM. **P< 0.01, unpaired Student’s *t* test (*n* = 7 mice)

While histopathology features of C9ALS/FTD (RNA foci and DPRs) are present throughout the brains of all four C9BAC mouse models [11–14], only two of these models develop hippocampal neurodegeneration [11, 14] and one exhibits spatial learning and memory deficits [11]. Here, we analyzed the nuclear staining pattern and intensity of H3K9me3 foci in hippocampal neurons using the anti-NeuN antibody (NeuN signal is not shown for easier visualization) in the C9BAC mouse model, which does not show significant hippocampal neuronal loss or phenotypic deficits [13]. Confocal images and quantification revealed that the mean intensity of H3K9me3 staining per nucleus was strongly reduced in neurons of the DG, CA1, and CA3 in the hippocampus of 9-month-old C9BAC mice compared with control mice (Fig. 5a, b); nonetheless, these less intensely stained H3K9me3 foci remained co-localized with NucBlue-positive chromocenters (Fig. 5a). Additionally, quantification of cell density showed a moderate but significant neuronal loss in the CA3 region of C9BAC mice, compared with control mice (Fig. 5c).

The loss of H3K9me3 in the hippocampus in addition to the neuronal loss in CA1, CA3, and DG prompted us to explore whether the C9BAC animals also display hippocampus-associated phenotypic deficits. We assessed the long-term (24 h) spatial memory in 6-month-old C9BAC and control mice, using the object location memory (OLM) task. OLM is a simple single-trial behavioral memory task, in which rodents are allowed to explore freely (avoiding stress) and rely on the rodent’s innate preference for novelty [49, 50]. Animals were presented with a non-displaced (ND) versus displaced (D) object in the OLM test (Fig. 5d). We found that, following 24 h of training, C9BAC mice performed poorly on the OLM test and displayed limited exploration of the displaced object relative to control mice (Fig. 5e, f). Together, these results show that neuronal loss and reduced H3K9me3 mark in C9BAC mouse hippocampus neurons are accompanied by a hippocampal-dependent deficit in spatial learning and memory.

## Discussion

We have used quantitative single-cell imaging to show that enrichment of H3K9me3, the classical epigenetic silencing mark present at chromocenters, is strongly reduced in several C9BAC brain cell types, including glia and (motor) neurons in the spinal cord, as well as in the motor cortex and hippocampus. The alterations of H3K9me3 in the hippocampus of C9BAC mice led us to identify previously undetected neuronal loss in CA3 and to also uncover hippocampus-dependent cognitive deficits. These results indicate that loss of the repressive epigenetic mark H3K9me3 is associated with neurodegeneration in the hippocampus and with corresponding cognitive impairments in the C9BAC mice. Our data, together with previous reports (discussed below), support a working model in which the H3K9me3 loss contributes to these pathological and behavioral alterations via aberrant gene transcription, a phenomenon that has been observed in C9ALS/FTD mouse models and patients.

To gain insights into disease mechanism(s) associated with C9ALS/FTD, several research groups have generated four C9BAC mouse models, all of which exhibit the characteristic molecular abnormalities, namely, RNA foci and DPRs, which are also observed in C9ALS/FTD patients [11–14]. Despite the consistent presence of these typical pathological features, the transgenic C9BAC mice do not all consistently develop neural loss and the clinical features found in human patients: two C9BAC mouse models exhibit neurodegeneration along with either motor [14] or cognitive and behavioral [11] deficits, but two other C9BAC mouse models do not display significant neuronal loss or phenotypic features [12, 13]. The reasons for these contrasting results are not known [51], but careful analyses and comparison of the different C9BAC models may help unraveling the molecular mechanisms that underlie the pathogenesis and phenotype of C9ALS/FTD. Here, for example, the moderate and region-specific neuronal loss (in CA3 but not in CA1 and DG) and in behavioral alterations (OLM) in the “non-symptomatic” C9BAC mouse model [13] were only detected because we analyzed the hippocampus in greater detail and uncovered a significantly reduced H3K9me3 nuclear staining. Our data show that C9BAC mice developed hippocampal neural loss and cognitive deficits without motor-associated deficits, suggesting that this mouse model recapitulates mild phenotypic features of FTD.

Here, we document that nuclear H3K9me3 staining is largely reduced in C9BAC astrocytes and neurons. Similar quantitative single-cell imaging studies of neurons in tissue derived from patients as well as in tau models of Alzheimer’s disease indicate a strong reduced nuclear signal for H3K9me3 [26] and H3K9me2 [28, 29]. Additional analysis by Mansuroglu and colleagues [26] revealed that reduced H3K9me3 labeling in neuronal nuclei from Alzheimer’s disease is accompanied by a strong presence of this mark in the cytoplasm. Hoechst staining showed that the structure of chromocenters in Alzheimer’s disease neurons is disrupted. In our study, however, imaging assays do not indicate that in C9BAC cells the H3K9me3 mark is redistributed from the nucleus to the cytoplasm or that cHC and nuclear integrity is compromised.

We also evaluated another brain region (striatum) and peripheral tissues (liver and testis) and observed no changes in H3K9me3 staining (Additional file 1: Figure S4-5). All the cells analyzed in the present study (i.e., astrocytes, neurons, hepatocytes, and Sertoli cells) exhibit different heterochromatin features as evidenced by the DAPI/NucBlue staining. Based on these results, we suggest that the loss of H3K9me3 is more likely associated to specific brain regions (cortex and hippocampus) rather than to the heterochromatin structure.

Our imaging and biochemical assays show that C9BAC astrocytes exhibit a strong decrease in total nuclear H3K9me3 levels and in H3K9me3 enrichment at chromocenters compared to that in control astrocytes. Interestingly, immunostaining analysis in poly-PR mice showed that GFP-conjugated PR protein (a 50 repeat PR protein) localized to the H3K9me3, H3K27me3, and H3K4me3 marks; the latter two marks were also increased in GFP-PR-positive cells [15]. Recent investigations using cell lines suggest that the expression of SOD1 and TDP43 could also contribute to global epigenome alterations [52]. These findings point to a need for further in-depth analysis of H3K9me3 alterations in brain cells expressing mutant C9orf72, SOD1, and TDP43. Despite these findings, we found that C9BAC astrocytes exhibit a strong decrease in total nuclear H3K9me3 levels. Esanov and collaborators, using of ChIP-qPCR assays on the same C9BAC mouse model, documented that H3K9me3 enrichment at the human *C9ORF72* gene promoter is significantly increased [53]. Other ChIP-qPCR assays, together with bisulfide sequencing of the expanded *C9ORF72* locus, performed on tissue and cells derived from C9-ALS/FTD patients, also show that the local epigenetic status is significantly altered, with an enrichment of the repressive H3K9me3 and H3K27me3 marks as well as DNA methylation [53–57]. These data collectively indicate that while C9BAC expressing cells exhibit a strong decrease in total nuclear H3K9me3 levels in astrocytes and neurons, at least some of the remaining H3K9me3 mark may be redistributed within the genome and be deposited at specific genes such as *C9ORF72.* The increased epigenetic silencing of the mutant *C9ORF72* allele by DNA hypermethylation and H3K9me3 is believed to protect neurons from the burden of the pathology associated with the repeat-expansion [54, 58, 59]. Based on these results, we speculate that an additional loss of nuclear H3K9me3 levels, due for example to aging or environmental factors, could jeopardize H3K9me3 enrichment on the mutant *C9ORF72* allele, and thereby facilitate and increase in the repeat-expansion expression associated with this pathology.

What are the functional consequences of a lower level of H3K9me3 in C9BAC brain cells? ChIP assays have documented H3K9me3 enrichment at transcriptionally silent protein coding genes in both heterochromatic and active euchromatic regions [50, 60–63]. These data indicate that reduced H3K9me3 in C9BAC cells might result in increased expression of genes, if other epigenetic mechanisms, such as DNA methylation, exchange of histone variants, nucleosome remodeling, and/or long non-coding RNAs, do not counterbalance the loss of the repressive histone mark. Whether such compensatory epigenetic mechanisms occur is presently unknown. However, if so, they appear to be insufficient at coding genes as widespread alterations in mRNA expression have been detected in the transcriptome of brain tissue [19], skin biopsy-derived fibroblasts [16, 17], and iPSCs [16, 18] from C9orf72 patients as well as in the spinal cord and cortex from C9BAC mice [12]. Lower levels of H3K9me3 at cHC regions could also have important deleterious consequences, as determined from studies of mice that are knockout for H3K9 methyl transferases. For example, Suv39h1/2-deficient mice display severely reduced viability, chromosomal instability (presence of hyper-tetraploid number of chromosomes), and higher levels of major satellite repeat transcripts that underlie the pericentromeric cHC regions [43, 44]. Furthermore, lower levels of H3K9me3 in C9BAC brain cells could influence the chromatin organization and gene expression of large continuous genomic regions that associate tridimensionally with chromocenters inside the nucleus [64]. That is, pericentromeric cHC regions have local chromatin interactions identified by Hi-C with other genomic regions that are sufficient to induce H3K9me3 deposition and to contribute to the repression of associated genes. Therefore, a decrease in H3K9me3 levels (in the absence of compensatory epigenetic mechanisms such as DNA methylation) could influence coding and non-coding gene expression in a trans and cis manner. Notably, evidence for aberrant expression of coding and non-coding genes, including repeat elements, has been reported in patients and in tau animal models of AD [28, 29, 65] that also display reduced H3K9me3 [26] or H3K9me2 staining signals [28, 29]. Interestingly, abnormal expression of repeat elements has also been reported in poly-PR mice [15]. Additional ChIP and transcriptomic analyses of C9BAC mice should establish whether aberrant coding and non-coding gene expression are associated with the depletion of H3K9me3 at specific genomic regions.

## Methods

### Animals

All protocols involving mice were carried out according to NIH guidelines and ARRIVE guidelines and were approved by the Ethical and Bio-security Committees of Universidad Andrés Bello. Transgenic mice line expressing a familial ALS/FTD human patient-derived C9orf72 genomic DNA sequence (C9BAC) [13] was a kind gift of Dr. Robert Brown (University of Massachusetts Medical School, Worcester) and B6SJLF/J mice were used as control subjects. Transgenes in the C9BAC mice were identified by PCR using the primers for Orf72e3: forward 5′ TTA ATT TCC TAC CCC TGC CC 3′ and reverse 5′ AGG CCT TGA CAA ATG TAG CC 3′ and for Mm10chr3: forward 5′ GCC TCA CCt CCT AAG AGC CTA 3′ and reverse 5′ CCT TTG TGT CAC ACG GAT ATC 3′ [13].

### Primary mouse spinal cord astrocytes and ACM preparation

The primary mouse spinal cord astrocyte cultures were generated from individual whole spinal cords of P1-2 wild-type mice and transgenic C9BAC mice, and PCR (see above) of tail tissue of each neonatal mouse was performed to define whether the astrocytes were from wild-type mice (Ctr) or transgenic C9BAC mice, as previously described [33, 34, 66]. Briefly, spinal cords were excised, minced, and enzymatically treated by incubating in pre-warmed PBS containing 0.25% trypsin (Gibco, Cat. No. 15090-046) for 20 min at 37 °C. Cells were maintained in DMEM (Hyclone, Cat. No. SH30081.01) containing 10% FBS (Gibco, Cat. No. 16000-044), 1% l-glutamine (Gibco, Cat. No. 25030-081), and 1% penicillin–streptomycin (Gibco, Cat. No. 15140-122) at 37 °C 5% CO_2_. Cultures reached confluence after 2–3 weeks and contained > 95% GFAP+ astrocytes. Residual microglia were removed by shaking cultures in an orbital shaker (200 rpm in the incubator) overnight, at which point media was replaced by neuronal growth media, including 70% MEM (Gibco, Cat. No. 11090-073), 25% Neurobasal media (Gibco, Cat. No. 21103-049), 1% N2 supplement (Gibco, Cat. No. 17502-048), 1% l-glutamine (Gibco, Cat. No. 25030-081), 1% penicillin–streptomycin (Gibco, Cat. No. 15140-122), 2% horse serum (Gibco, Cat. No. 15060-114; lot 1517711), and 1% sodium pyruvate (Gibco, Cat. No. 11360-070). After 7 days, ACM was collected, supplemented with 4.5 mg ml^−1^d-glucose (final concentration), and filtered and stored at – 80 °C. These media were used to evaluate survival of motoneurons in primary ventral spinal neuronal cultures prepared from Sprague–Dawley rats at E14 [33, 34, 66].

### Immunostaining

The whole immunostaining process, including the incubation with the specific antibodies, was always carried out in parallel in both control and C9orf72 cells/tissues. Paraformaldehyde-fixed cultured astrocytes, brain tissue sections, testicular cells, and liver hepatocytes from control and C9BAC mice were used for immunostaining experiments. Astrocyte cultures (see above) were fixed with fresh 4% paraformaldehyde for 20 min, permeabilized with 0.1% Triton X-100 in PBS for 20 min, and incubated with normal goat serum (10%) blocking solution (Invitrogen, 50062Z) for 30 min. Brain tissue sections were incubated with blocking/permeabilization solution (3% donkey serum and 3% BSA in 0.5% Triton X-100 in PBS) for 4 h. For tissue sectioning, the brain and spinal cord were carefully removed from transcardially perfused mice and immersed in 4% paraformaldehyde overnight. Samples were cryoprotected in 30% sucrose and sliced sagittally (40 μm thick) using a cryostat (Leica CM1520). Testicular cells were obtained by performing cellular spreads isolating the seminiferous tubules and mechanically disaggregating using forceps. Then, 200 μl of 100-mM sucrose was slowly added and mixed with the cells. From this suspension, 14 μl was dropped onto a slide previously submerged in 1% paraformaldehyde (PFA), pH 9.2, and spread throughout the slide. Next, the slides were slowly dried in a humid chamber for 3 h, washed with Photo-Flo 0.08 % in distilled water, air-dried, and stored at – 80 °C until their use. Sertoli cells were identified and classified based on the nuclear morphology (nuclear size and shape, as well as localization, distribution, and size of the chromocenters) observed with DAPI staining [67]. Liver hepatocytes were obtained by imprint cytology. Briefly, 4% paraformaldehyde-fixed liver was freshly cut, and liver imprints were made by gently touching the cut surface of the node onto a glass slide.

Cells and tissue sections were incubated overnight at 4 °C with primary antibodies: H3K9me3 (Abcam, USA Cat. No. ab8898, 1:1,000, rabbit), H3K9me2 (Abcam, USA, Cat. No. ab1220, 1:500, mouse), H3K9me1 (Abcam, USA, Cat. No. ab9045, 1:1000, rabbit), NeuN (Millipore, USA, Cat. No. MAB377, 1:300, mouse), and GFAP (DAKO, USA, Cat. No. Z0334, 1:1,000, rabbit). After rinsing three times with 0.5% Triton X-100 in PBS, cells and tissue samples were incubated with Alexa-conjugated secondary antibodies [Alexa Fluor 488 (Alexa^488^) or 546 (Alexa^546^), Invitrogen, USA] for 1 h at room temperature. ProLong Gold Antifade Mountant with DAPI (Thermo Fisher, Cat. No. P36931) or NucBlue (Invitrogen, Cat. No. R37605) were used to stain the nucleus in cells and in tissue sections, respectively.

Acquisition settings were always the same for the specific cell types, and acquisition was carried out in parallel in both control and C9orf72 cells/tissues. Images of primary astrocytes were taken on a Leica TCS SP8 confocal microscope with a × 63 oil objective and × 2 digital zoom (NA = 1.4; HC PL ACS APO) and a z-step of 0.5-μm optical sections (velocity scan 600 Hz; resolution 1024 × 1024 pixels, equivalent to 87.3 μm × 87.3 μm). The following laser wavelengths were used to detect DAPI (Ex 405 nm and Em 410–483 nm) and HPTMs-Alexa^546^ (Ex 561 nm and Em 570–625 nm). Maximum intensity projections of confocal z-stack images of whole nuclei (containing 10–12 stacks) were analyzed. Images of brain tissue sections were taken on an Olympus FV1000 confocal microscope with a × 60 oil objective (NA = 1.35; UPLSAPO) and a z-step of 0.5-μm optical sections (velocity scan 12 μs/pixel; resolution 1024 × 1024 pixels, equivalent to 211.761 μm × 211.761 μm). The following laser wavelengths were used to detect NucBlue (Ex 405 nm and Em 422–475 nm), cell type marker-Alexa^488^ (Ex 488 nm and Em 500–530 nm) and HPTMs-Alexa^546^ (Ex 543 nm and Em BA560–660 nm). Maximum intensity projections of confocal z-stack images of whole nuclei (containing 5–7 stacks in the spinal cord, 15–20 stack in the motor cortex, and 5–7 stacks in the hippocampus) were analyzed. Images of testicular cells and hepatocytes were obtained using a Nikon Eclipse E400 epifluorescence microscope equipped with an Infinity 5 camera. For these cell types, all the pictures were taken using a × 100 magnification lens.

The Fiji ImageJ software (8 bits, measuring intensity from 0 to 255) was used to measure the DAPI/NucBlue and H3K9me3 fluorescence intensity and mean nuclear area. Line scans were drawn in ImageJ to quantify the relative intensities of the fluorescence signal for DAPI and H3K9me3. The quantification of foci (≥ 0.50 μm) was carried out under threshold conditions using the analyze particle plugin. Maximum intensity projections of confocal z-stack images of whole nuclei were analyzed. At least 20 isolated nuclei were analyzed for each cell type (astrocytes, neurons, hepatocytes, and Sertoli cells). The number of neurons in hippocampal DG, CA1, and CA3 regions was counted as previously described [11] in 2–3 consecutive sections. Careful matching of the sections to compare similar anatomical regions was performed for each set of mice.

### Western blotting

Cultured astrocytes from control and C9BAC mice were lysed in an ice-cold lysis buffer [60 mM KCl, 15 mM NaCl, 2 mM EDTA, 0.5 mM EGTA, 15 mM Tris-HCl pH 7.4, 0.5 mM spermidine (Sigma-Aldrich, Cat. No. S2501), 0.5 mM DTT, and 0.2% nonidet NP-40]. Using the Kimble Dounce tissue grinder set (Sigma D8938), the membrane was disintegrated to obtain isolated nuclear fractions. The pellet was resuspended in a sonication buffer [50 mM HEPES pH 7.9, 140 mM NaCl, 1 mM EDTA, 1% Triton X-100, 0.1% sodium deoxycholate, and 10% SDS with protease inhibitors (Roche, Cat. No. 11836153001)] and sonication was performed by a Bioruptor (Diagenode) for 5 min at maximum power. Protein samples (10–20 μg) were resolved by 10% SDS-PAGE, transferred to a nitrocellulose membrane, blocked with 5% milk in PBS-Tween 20 0.1% (TPBS), and incubated overnight at 4 °C with rabbit anti-H3K9me3 (Abcam, USA Cat. No. ab8898, 1:1,000). After washing, the membrane was incubated with HRP-conjugated secondary antibody (Santa Cruz Biotechnology, 1:5,000) and developed using the ECL plus western blotting detection reagent from Thermo Scientific (Rockford, IL, USA). Then, for the detection of total H3, the same membrane was incubated with the ReBlot Plus Strong Antibody Stripping Solution (Millipore, Cat. No. 2504) for 3 min. After washing three times with 0.05% Tween, the membrane was blocked with 5% milk in TPBS for 30 min incubated overnight at 4 °C with mouse anti-H3 pan (Diagenode, Cat No. C15200011, 1:10000) and then incubated with HRP-conjugated secondary antibody (Santa Cruz Biotechnology, 1:5,000) and developed using the ECL technique. Densitometric analysis was performed using the ImageJ software. H3K9me3 was normalized to total H3 levels.

### Object location memory (OLM)

The OLM test was performed as previously described [50]. Briefly, the single trail OLM test consisted of three steps: habituation (5 min), a training session (10 min), and a test session (10 min) 24 h later. Mice were individually habituated in an apparatus that contained a cage inside an insonorized chamber. In the training session, the cage contained two identical objects, termed “none-displaced objects” (ND). In the test session, the location of one of the none-displaced objects was changed, termed displaced object (D). The exploration time was recorded and defined as time spent sniffing or touching the object with the nose and/or forepaws. The “discrimination index” was calculated by the time spent to explore object ND compared with total time explored in both objects (D + ND).

### Statistical analyses

Statistical analysis was performed using an unpaired Student’s *t* test (normal data) and a Mann–Whitney test when two populations were examined. A one-way ANOVA followed by the Bonferroni post hoc was utilized when making multiple comparisons. In all figures, error bars represent the SEM, *P < 0.05, **P < 0.01, ***P < 0.001.

## Supplementary information

**Additional file 1: Figure S1.** ACM derived from C9BAC astrocytes significantly reduces motoneuron survival. **Figure S2.** Loss of H3K9me3 staining in N2A cells treated with chaetocin. **Figure S3.** H3K9me3 staining overview in primary astrocytes cultured reveals global reduction of H3K9me3 fluorescence intensity. **Figure S4.** H3K9me3 staining in striatal neurons from C9BAC and control mice. **Figure S5.** H3K9me3 staining in hepatocytes and Sertoli cells from C9BAC and control mice.

## Data Availability

All data generated or analyzed during this study are included in this published article.

## Abbreviations

ACM: Astrocyte-derived conditioned media
ALS: Amyotrophic lateral sclerosis
BAC: Bacterial artificial chromosome
BSA: Bovine serum albumin
C9BAC: C9ALS/FTD BAC mouse model
CA: Cornu ammonis
cHC: Constitutive heterochromatin
D: Displaced
DG: Dentate gyrus
DPR: Dipeptide-repeat
FBS: Fetal bovine serum
fHC: Facultative heterochromatin
FTD: Frontotemporal dementia
GFAP: Glial fibrillary acidic protein
iPSC: Induced pluripotent stem cell
ND: Non-displaced
NeuN: Neuronal nuclei
OLM: Object location memory
PR: Proline-arginine
RAN: Repeat-associated non-AUG-dependent
