# Supplementary material for: Widespread loss of the silencing epigenetic mark H3K9me3 in astrocytes and neurons along with hippocampal-dependent cognitive impairment in C9orf72 BAC transgenic mice
Source: Clin Epigenetics. 2020 Feb 18;12:32. doi: 10.1186/s13148-020-0816-9 (PMC7029485; doi:10.1186/s13148-020-0816-9)
Supplement: Supplementary file 1 — Additional file 1: Figure S1. ACM derived from C9BAC astrocytes significantly reduces motoneuron survival. Figure S2. Loss of H3K9me3 staining in N2A cells treated with chaetocin. Figure S3. H3K9me3 staining overview in primary astrocytes cultured reveals global reduction of H3K9me3 fluorescence intensity. Figure S4. H3K9me3 staining in striatal neurons from C9BAC and control mice. Figure S5. H3K9me3 staining in hepatocytes and Sertoli cells from C9BAC and control mice. [file 13148_2020_816_MOESM1_ESM.pdf]

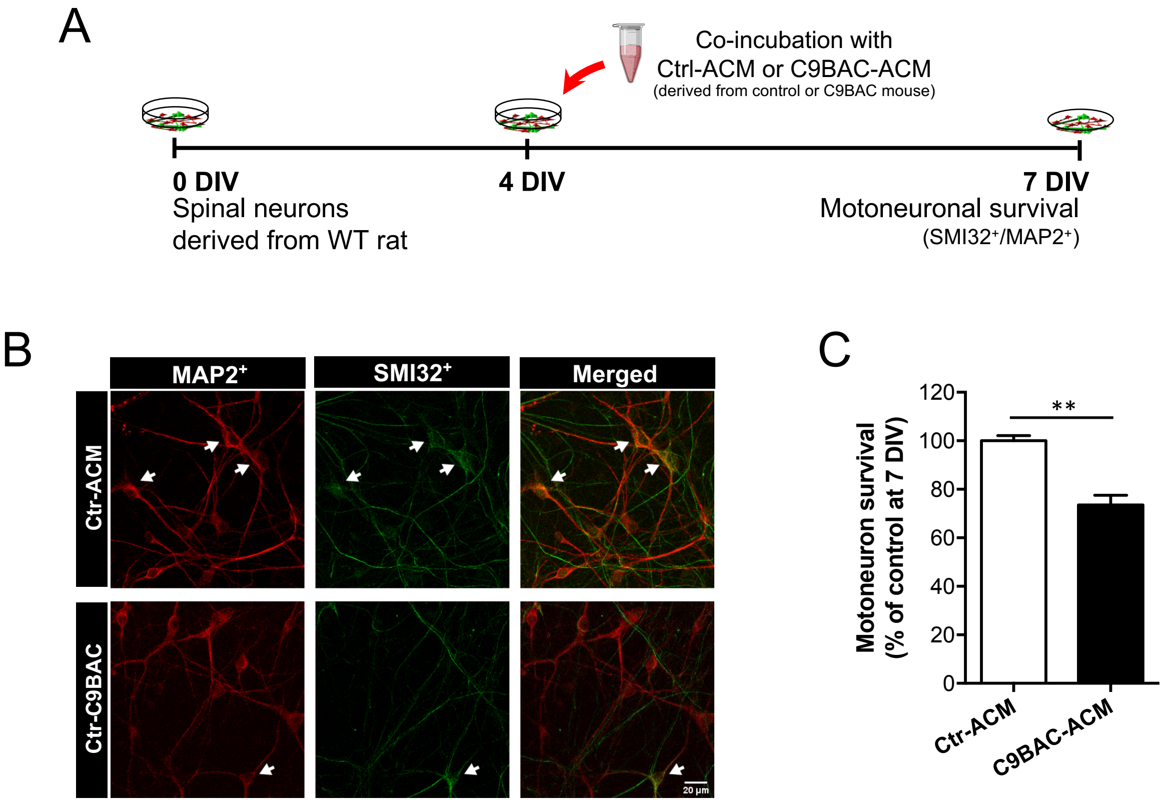

Supplementary figure 1: ACM derived from C9BAC astrocytes significantly reduces motoneuron survival. (A) Flow diagram of experiment. After 4 days *in vitro* (DIV), primary wild-type (WT) rat spinal cord cultures were exposed for 3 days to astrocyte-conditioned media (ACM) generated from either C9BAC (C9BAC-ACM) or control (Ctrl-ACM) primary astrocyte cultures. At 7 DIV, spinal cord cultures were fixed and cell survival was assayed by immunostaining, using an anti-microtubule-associated protein 2 (MAP2) antibody to visualize interneurons plus motoneurons, and an anti-SMI-32 antibody to identify only motoneurons. **(B)** Representative images showing $MAP2^+/SMI32^+$ labeled neurons (white arrows) in rat spinal cord cultures treated with Ctrl-ACM or C9BAC-ACM. **(C)** Quantification of the percentage of motoneuron survival ($SMI32^+/MAP2^+$ cells) exposed to Ctrl-ACM or C9BAC-ACM. Bars represent mean±S.E.M. $^{**}P<0.01$, unpaired Student's *t*-test (n=3 independent experiments).

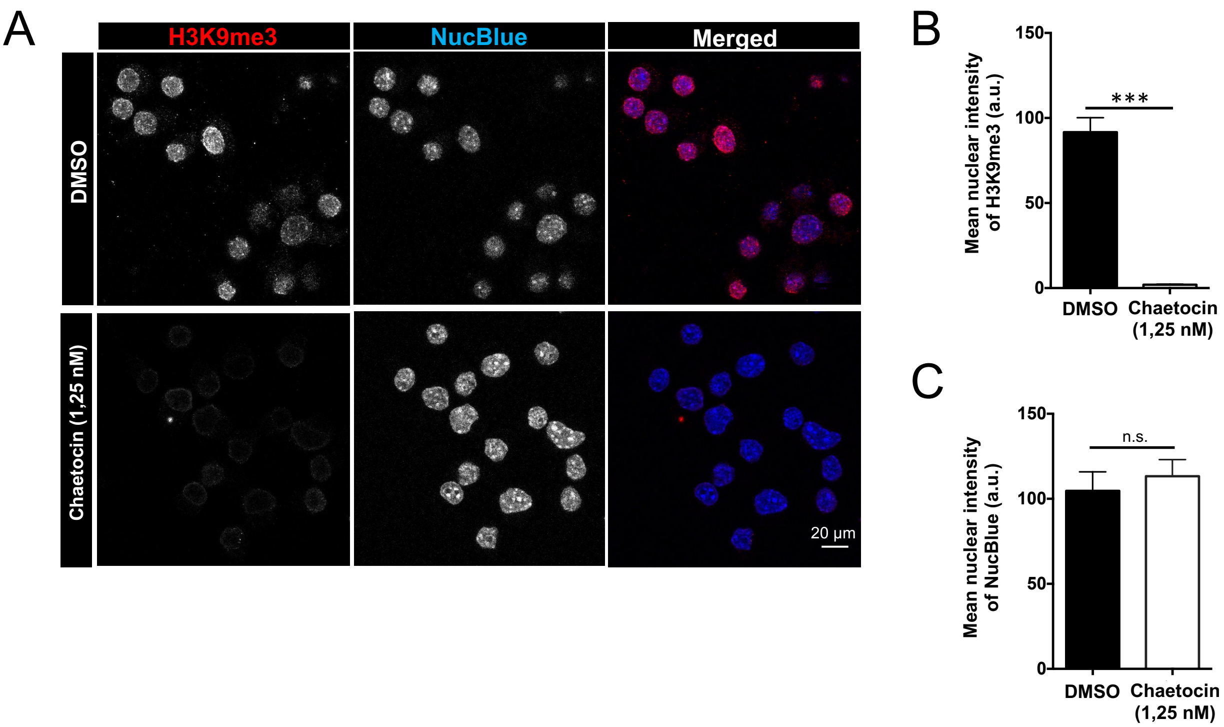

Supplementary figure 2: Loss of H3K9me3 staining in N2A cells treated with chaetocin.

(**A**) Representative confocal images of the immunofluorescence staining of H3K9me3 (red) and NucBlue (blue) in N2A cells in the absence or presence of chaetocin (1,25 nM for 24 h), a pharmacological inhibitor of H3K9 methyltransferase SUV39H1/2. (**B-C**) Quantification of the mean nuclear intensity of H3K9me3 (**B**) and NucBlue (**C**) staining, shown as arbitrary units (a.u.). In both graphs, bars represent mean±S.E.M. ***P<0.001; non-statistical differences (ns), Student's t-test (n=3 independent experiments).

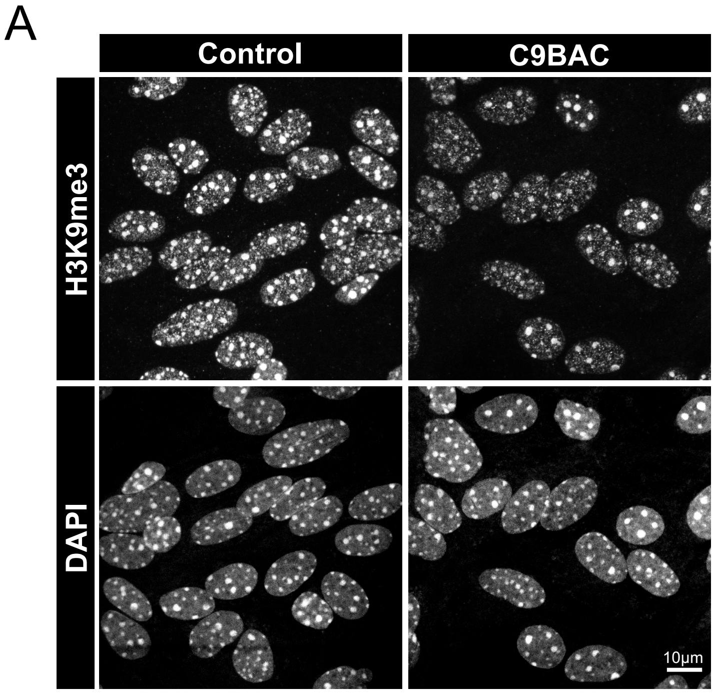

Supplementary figure 3: H3K9me3 staining overview in primary astrocytes cultured reveals global reduction of H3K9me3 fluorescence intensity. Lower magnification of images shown in Figure 1A. Representative confocal images of the immunofluorescence staining of H3K9me3 in primary cultures of control and C9BAC astrocytes. Nuclei are stained with DAPI. The images represent a maximum projection for the total nuclear volume.

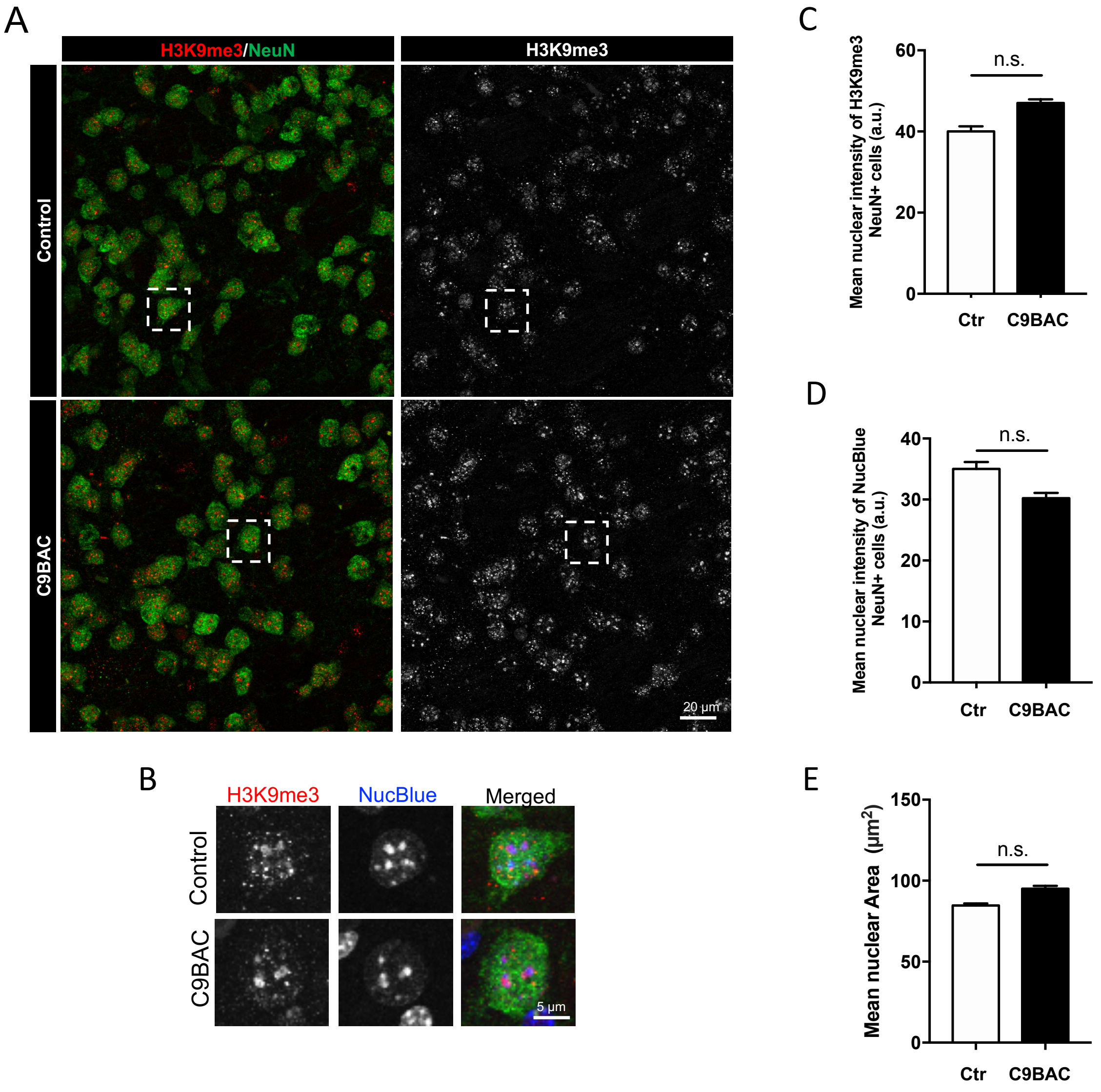

Supplementary figure 4: H3K9me3 staining in striatal neurons from C9BAC and control mice. (**A-B**) Representative confocal images of immunofluorescence staining for H3K9me3 (red and white) and NeuN (green) showing individual and merged images of the striatum of control and C9BAC mice (coronal brain section 40 µm). The images represent a maximum projection for the total nuclear volume. (**C-E**) Quantification of the mean nuclear intensity of H3K9me3 (**C**) and NucBlue (**D**) staining, shown as arbitrary units (a.u.), and mean nuclear area (**E**). Bars represent mean±SEM. Non-statistical differences (ns), Student's *t*-test (n=3 independent experiments).

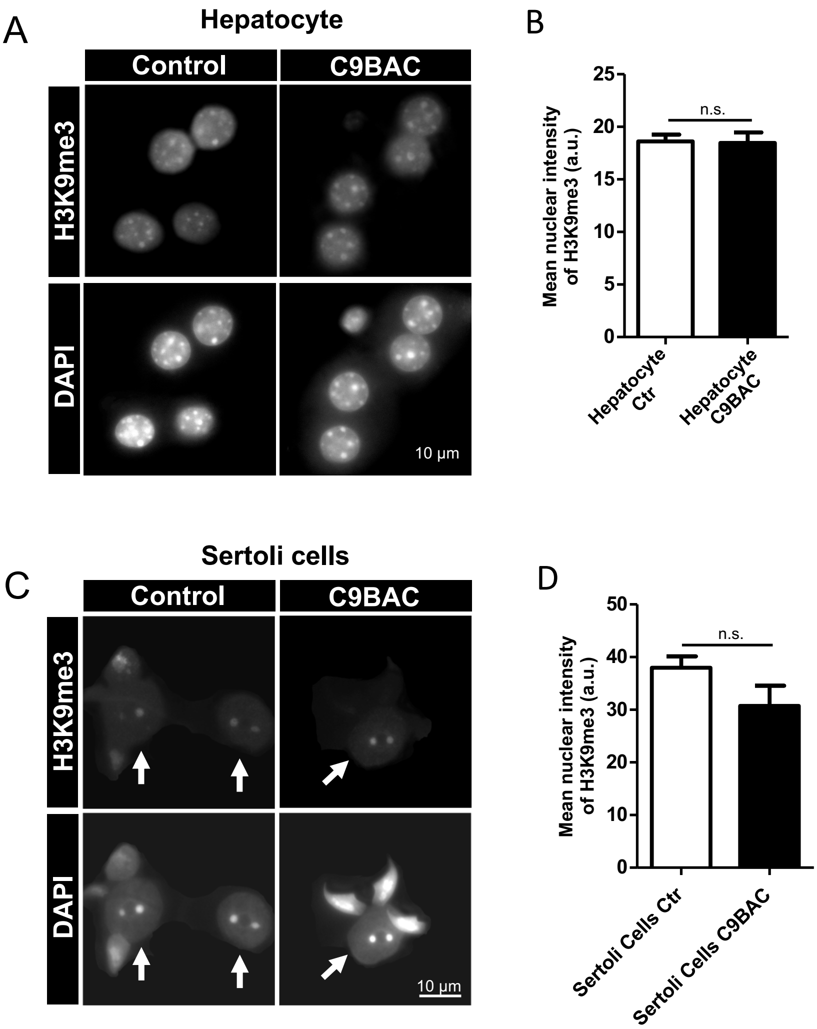

Supplementary figure 5: H3K9me3 staining in hepatocytes and Sertoli cells from C9BAC and control mice. (**A**) Representative confocal images of immunofluorescence staining for H3K9me3 and DAPI in hepatocytes from control and C9BAC mice. The images represent a maximum projection for the total nuclear volume. (**B**) Quantification of the mean nuclear intensity of H3K9me3, shown as arbitrary units (a.u.). (**C**) Representative confocal images of immunofluorescence staining for H3K9me3 and DAPI in Sertoli cells isolated from testis from control and C9BAC mice. The images represent a maximum projection for the total nuclear volume. (**D**) Quantification of the mean nuclear intensity of H3K9me3 (shown a.u.) in Sertoli cells. Bars represent mean±SEM. Non-statistical differences (ns), Student's *t*-test (n=3 independent experiments).
